# Building antimicrobial stewardship through massive open online courses: a pilot study in Macedonia

**DOI:** 10.1093/jacamr/dlaa045

**Published:** 2020-07-18

**Authors:** Sebastian von Schreeb, Elizabeth Robilotti, Stan Deresinski, Golubinka Boshevska, Nikola Panovski, Mia Tyrstrup, Katarina Hedin, Neda Milevska-Kostova

**Affiliations:** 1 Centre for Regional Policy Research and Cooperation “Studiorum”, Nikola Parapunov 41, 1000 Skopje, Macedonia; 2 Department of Medicine, Division of Infectious Diseases and Geographic Medicine, Stanford University School of Medicine, 300 Pasteur Drive, Stanford, CA 94305, USA; 3 Institute of Public Health, 50 Divizija 6, Skopje, Macedonia; 4 Institute of Microbiology and Parasitology, Medical Faculty, University “Ss. Cyril and Methodius”, Vodnjanska 17, 1000 Skopje, Macedonia; 5 Department of Clinical Sciences in Malmö, Family Medicine, Lund University, Malmö, Sweden

## Abstract

**Background:**

The global struggle against antibiotic resistance requires antimicrobial stewardship (AMS). Massive open online courses (MOOCs) offer health professionals unprecedented access to high-quality instructional material on AMS; the question is how apprehensible it is to non-native English speakers. Furthermore, to better understand how education interventions promote change towards rational antibiotic prescribing, leading institutions call for studies integrating behavioural science. Research from lower- and middle-income countries is particularly needed.

**Objectives:**

To measure the knowledge improvement from an AMS MOOC, the influence of language, course satisfaction and subsequent effect on intention to change antibiotic prescribing behaviour.

**Methods:**

Fifty-five physicians from Macedonia completed the MOOC. Pre- and post-course knowledge test scores were compared using a one-sample *t*-test. The effect of a language barrier was assessed using self-reported English level. Scores were compared with participants’ intention to change behaviour in clinical practice.

**Results:**

Scores significantly improved from 77.8% to 82.2%. Participants with a higher English level improved most, while the low-level group showed no significant improvement. Physicians reported a high or very high intention to change behaviour. This was independent of knowledge improvements.

**Conclusions:**

First, lower self-reported English proficiency hindered knowledge acquisition from a MOOC platform. AMS programmes should commit to bridge this barrier so as to enable a global spread of education in AMS. Second, factors underlying the physicians’ intentions to engage in AMS appear to be more complex than simple knowledge improvements. This suggests that less time-consuming interventions could be as effective.

## Introduction

Antimicrobial resistance (AMR) is one of the largest public health threats of our time.^1^ If action is not taken, the world is heading towards a post-antibiotic state in which minor infections once again kill. Over the last two decades, global consumption of antibiotics has risen by one-third, which is largely attributable to irrational use.^2^

A key action to combat this development is to build capacity in antimicrobial stewardship (AMS)^3^ to ensure that antimicrobials are prescribed in line with clinical guidelines.^4^

Optimizing prescriptions entails facilitating behaviour change among prescribers.^1^ In the Global Action Plan on AMR, WHO declares a need for scientific studies on mechanisms underlying behavioural change among health professionals.^1^

In this area of research, most studies are set in high-income countries.^5^ To face the issue of generalizability and broaden the geographical context of this research, more studies from both low- and middle-income countries (LMICs) are needed.^5^

From a global perspective, it is particularly important to build AMS capacity in LMICs, as these countries face a larger AMR burden.^6^ In the EU, the increase in antibiotic use is largest in southern and eastern countries, while some northern countries have documented a decrease.^7^ In Macedonia, a middle-income country in southeast Europe, antibiotic consumption is estimated to be twice as high as the average European level.^8^^,^^9^ Evidence suggests that it is possible to safely reduce antimicrobial prescribing.^2^

### Online education

Educating health professionals is the most commonly applied AMS intervention. Education constituting the foundation for evidence-based prescribing allows professionals to assist in disease prevention and control, to advise patients in rational antibiotic use and to advocate for a public responsibility to combat AMR.^10^

The traditional model of medical education and continuous medical education (CME) is in transition.^11^^,^^12^ In recent years, there has been a vast expansion of massive open online courses (MOOCs)—web-based, self-paced courses often developed by world-leading academic institutions, allowing a very large number of participants.^13^ They appear to be at least as effective as traditional teaching methods in medical education and offer an unprecedented level of accessibility.^14–16^

Several high-quality AMS online courses aimed at healthcare professionals already exist and this material could be leveraged to achieve wider dissemination of AMS training.^17^ Most of these courses have been created by and for native English speakers, presenting a potential obstacle for broad deployment of these teaching materials.

This raises the question of a potential language barrier preventing knowledge dissemination of AMS, which has been discussed but not sufficiently explored.^18^ When doctors are non-native English speakers, the usefulness of existing MOOCs may be altered or lessened.^19^ As translating MOOC material is a high resource-demanding process, there is need to better map the language barrier.^17^

### Changing behaviour

Although education is the most commonly employed AMS intervention, it is only marginally effective in causing behaviour change.^20^^,^^21^ Rather, in behavioural science theory, education is seen as one of many factors—personal, social and environmental—that influence behaviour.^22^

The need to integrate behavioural science into AMS interventions was stressed in a recent Cochrane review, which concluded that this potential is still underutilized.^23^ This is also the case in LMICs.^24^ A theoretical understanding of factors underlying human behaviour helps to uncover the wide spectrum of possible health interventions and to maximize their potential efficacy.^12^^,^^25^ In the broader realm of health research, the last two decades have seen an expansion of behavioural change techniques, reviewed by Michie *et al*.^26^ and classified by the Cochrane EPOC group.^27^

While it is certain that antibiotic prescribing in LMICs is excessive, it is not clear to what extent prescribers lack knowledge in AMS. It has been shown that education interventions have an effect,^23^ but it is not clear if this is mediated through an improvement in knowledge. The relationship between knowledge improvements and behavioural change should be investigated in order to optimize education interventions.

How can behaviour change be measured? Previous research has established that a person’s intention to change behaviour is a good predictor of subsequent behaviour change.^28^^,^^29^ Furthermore, research has shown that while people’s intentions better predict how they will act, their estimation of future behaviour, considering potential barriers to change, more readily predicts goal attainment.^30^ Therefore, to increase validity, both the participants’ intentions to change and their estimations of success should be considered.

To summarize, there is a need for studies from middle-income countries that investigate online AMS education. It is not clear to what extent MOOCs can improve knowledge in AMS and whether this is hindered by language proficiency. Finally, this needs to be linked to measures of behavioural outcome and integrated with the body of knowledge constituted by behavioural science.

In light of the presented knowledge gaps, the current study set out to assess the effects of a MOOC on AMS among health professionals in Macedonia. The research questions were: (i) To what extent does participation in an online course on AMS improve knowledge? (ii) Is knowledge improvement hindered by a language barrier? (iii) Does the target group perceive an online course on AMS to be an acceptable mode of education? and (iv) Does participation in online education promote the prescriber’s intention to change antibiotic prescribing behaviour? Collaborating institutions were the WHO Regional Office for Europe, Stanford University, University “Ss. Cyril and Methodius” and the Centre for Regional Policy Research and Cooperation “Studiorum”.

## Methods

### Study population

An invitation was sent to all 70 secondary- and tertiary-level public and private healthcare facilities in Macedonia, requesting sign-up of up to five participants interested in taking the MOOC.

### Tool development and procedure

#### MOOC

The original MOOC, ‘Antimicrobial Stewardship: Improving Clinical Outcomes by Optimization of Antibiotic Practices’, by Stanford University is offered free of charge through the online platform Coursera and Stanford Medicine CME Center (https://online.stanford.edu/courses/som-ycme0001-antimicrobial-stewardship-improving-clinical-outcomes-optimization-antibiotic). Participants who complete all 26 modules and pay a fee of US$20 are awarded six CME credits through Stanford Medicine CME Center.

**Figure 1.**
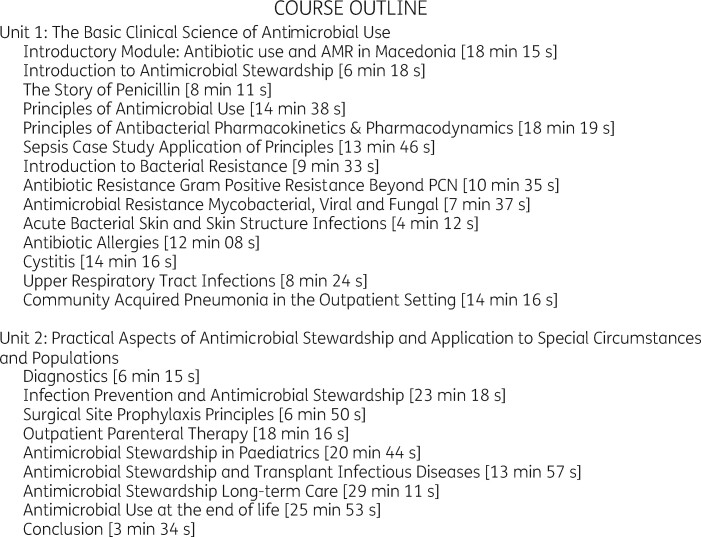
Modules included in the MOOC on AMS adapted for Macedonia. Duration of video for each module is shown in brackets.

In the current study, the MOOC was adapted to the context of Macedonia, excluding US-specific modules, and adding a new introductory module developed to fit the national healthcare setting (Figure 1).

#### Surveys

Four surveys were administered: two knowledge assessments, one satisfaction survey and a CME test. Each survey included participant characteristics, including self-reported English level categorized on four skills: reading, writing, speaking and listening.

The knowledge assessment, provided before and after the course, consisted of 58 multiple-choice questions divided into three main categories: (I) awareness and knowledge; (II) prescribing competency; and (III) managing infections (Appendix 1, available as Supplementary data at *JAC-AMR* Online). It was developed by Stanford University, based on its medical curriculum and amended with questions related to central topics from each course module. The satisfaction survey was constructed using the Coursera participant survey as well as participant satisfaction surveys used for WHO courses and seminars (Appendix 2). The questionnaire was reviewed by subject experts from WHO.

To increase motivation and reduce dropout, participants were offered a CME accreditation at no cost to the participant upon successful completion of the CME test. To reduce response bias, the order of questions within categories was shuffled. To increase confidentiality, data from each survey were anonymized.

#### Pilot

Prior to initiating the study, the knowledge assessment was piloted among a group of Danish medical students. When respondents’ interpretations differed, questions were rephrased or dropped from the instrument. Items with more than 10% missing responses were excluded from the final version of the survey.

#### Procedure

Upon completing the pre-course knowledge assessment, participants were provided with login credentials to the password-protected course webpage. They were given 4 weeks to complete the course through self-study. Participants then took the post-course knowledge assessment, the CME test and the satisfaction survey, accessed through links provided via e-mail. During the period of the study, four reminder e-mails were sent. The intervention took place between 24 April 2015 and 19 August 2015.

### Statistical analysis

#### Reliability and validity

Within 2 weeks of administering the post-course knowledge assessment, a subsample of respondents was randomly selected for a test–retest reliability analysis. Responses were considered reliable if the correlation between surveys was 0.7 or higher.^31^ Validity testing involved two steps: (i) pilot testing with instrument revision; and (ii) exploratory factor analysis. The factor analysis assessed how different items aligned into common constructs that describe different elements of AMS competencies.^32^

#### Correct answers

Participants received one point per correct answer and one point per correct response for questions with multiple correct alternatives. In total, the maximum score was 89 points.

#### Knowledge improvement

A one-sample *t*-test was used to evaluate the difference between scores on the pre-course and post-course knowledge assessment test. This statistical method is recommended when results are anonymized so that individual participants cannot be identified.^33^ To examine violations of normality, a Shapiro–Wilks test was used. Participants completing less than 10% of the test were considered to have dropped out.

#### Language barrier

In order to evaluate the effect on knowledge improvement, participants’ English level was dichotomized into a high- and a low-level group. The high-level group consisted of participants who reported having a professional working proficiency or higher. The low-level group were those reporting to have limited, elementary or no English proficiency. Knowledge score change was evaluated for each group using a one-sample *t*-test.

### Ethics

The Ethics Committee of the Medical faculty in Skopje does not require ethics approval for this kind of intervention. However, prior to enrolment, all participants were asked to complete an online course registration form and to complete and sign a ‘Statement of Ethical Conduct in Academic Communities on the Internet’.

## Results

### Participants

Seventy-four participants responded to the initial invitation, of which 55 self-registered for the online course and completed the required ethical conduct form. Due to dropout, a total of 51 and 38 persons completed the pre-course and post-course knowledge assessment, respectively, and 28 participants finished the satisfaction survey. At baseline, 45.5% reported to have actively participated in a stewardship programme at work, while 53% had not (one missing). Eighteen percent had participated in AMS training in the 12 months preceding the survey. In total, 80% of respondents encountered patients with bacterial infections daily to weekly in their clinical practice and 60% reported prescribing antibiotics at least five times per work week. Table 1 shows characteristics of the study population. In total, 74.5% of participants were women and the mean age was 43.9 years, with a standard deviation of 9.5 years.

**Table 1.** Characteristics of the study population in a MOOC on AMS

Characteristics of study population	Pre-course survey, *N *=* *51	Post-course survey, *N *=* *38	Satisfaction survey, *N *=* *28
	*n* (%)	*n* (%)	*n* (%)
Gender			
female	39 (76.5)	27 (71.1)	20 (71.4)
male	12 (23.5)	11 (28.9)	8 (28.6)
Age (years)			
25–34	8 (15.7)	5 (13.2)	5 (17.9)
35–44	18 (35.3)	13 (34.2)	10 (35.7)
45–54	17 (33.3)	15 (39.5)	7 (25.0)
55–64	8 (15.7)	5 (13.2)	6 (21.4)
Workplace			
hospital	29 (56.9)	21 (55.3)	no data
primary care	9 (17.6)	7 (18.4)	no data
academic institute	10 (19.6)	6 (15.8)	no data
public health institute	3 (5.9)	4 (10.5)	no data
Medical speciality			
paediatrics	9 (17.6)	4 (10.5)	7 (25.0)
internal medicine	5 (9.8)	5 (13.2)	3 (10.7)
gynaecology and obstetrics	4 (7.8)	2 (5.3)	0 (0)
general practice and family medicine	9 (17.6)	6 (15.8)	6 (21.4)
surgery	3 (5.9)	2 (5.3)	1 (3.6)
urology	2 (3.9)	2 (5.3)	1 (3.6)
infectious diseases	5 (9.8)	4 (10.5)	5 (17.9)
medical microbiology	7 (13.7)	6 (15.8)	3 (10.7)
other^a^	7 (13.7)	7 (18.4)	2 (7.1)

Demographics are shown for the number of participants finishing the pre-course assessment (51) post-course assessment (38) and satisfaction survey (28).

a Other* *=* *immunology, clinical pharmacy, anaesthesiology, forensic medicine and criminalistics.

### Knowledge improvement

Participants correctly answered 77.8% of questions in the pre-course knowledge assessment, with an SD of 8.1 percentage points (pp) (Table 2).

**Table 2.** Summary of results

Summary of results (number of questions)	Pre-course (*n *=* *51), % (SD)	Post-course (*n *=* *38), % (SD)	Change (pp)
I. Awareness and knowledge (39)	82.4 (7.1)	84.4 (8.9)	2.0
bacteria (5)	76.9 (18.1)	81.1 (15.4)	4.0
antibiotics (14)	81.7 (10.9)	83.3 (11.6)	1.6
bacterial resistance (15)	76.3 (9.9)	79.7 (13.1)	3.4
infection prevention and control (5)	93.3 (9.6)	93.0 (11.1)	−0.3
II. Prescribing competency (14)	71.1 (12.6)	77.9 (13)	6.8
patient safety (6)	74.2 (16)	80.2 (14)	6.0
diagnosis and indication (8)	65.3 (15.7)	83.8 (20.5)	18.5
III. Managing infections (5)	69.3 (16.4)	79.5 (14.8)	10.2
Total (58)	77.8 (8.1)	82.2 (9.4)	4.4

Results are shown as percentage of correct answers (SD), so that 100% would be correct answers to all questions.

Participants’ results in the pre- and post-course assessment were compared using a one-sample *t*-test. A Shapiro–Wilks test of the post-course scores did not show violation of normality, F(38)* *=* *0.95, *P *=* *0.08. Compared with the pre-course mean of 77.8%, the mean post-course score, 82.2%, was significantly higher, t(37)* *=* *2.86, *P *=* *0.007, CI = 0.79 to –0.85.

The highest improvement was seen in the survey category ‘(III) managing infections’, in which participants’ knowledge increased by 10.2 pp, followed by ‘(II) prescribing competency’ (6.8* *pp) and ‘(I) awareness and knowledge’ (only 2* *pp) (Table 2).

### Language barrier

Table 3 shows self-reported English level in the two knowledge assessments and the satisfaction survey.

**Table 3.** Participants**’** self-reported English proficiency level

English level, self-reported	Pre-course test, *n *=* *51	Post-course test, *n *=* *38	Satisfaction survey, *n *=* *28
	*n* (%)	*n* (%)	*n* (%)
Reading			
native or bilingual proficiency	4 (7.8)	1 (2.6)	0 (0)
professional working proficiency	32 (62.7)	23 (60.5)	16 (57.1)
limited working proficiency	11 (21.6)	9 (23.7)	8 (28.6)
elementary proficiency	4 (7.8)	5 (13.2)	4 (14.3)
no proficiency	0 (0)	0 (0)	0 (0)
no response	0 (0)	0 (0)	0 (0)
Writing			
native or bilingual proficiency	3 (5.9)	1 (2.6)	1 (3.6)
professional working proficiency	23 (45.1)	17 (44.7)	13 (46.4)
limited working proficiency	17 (33.3)	12 (31.6)	8 (28.6)
elementary proficiency	6 (11.8)	8 (21.1)	6 (21.4)
no proficiency	0 (0)	0 (0)	0 (0)
no response	2 (3.9)	0 (0)	0 (0)
Speaking			
native or bilingual proficiency	3 (5.9)	1 (2.6)	0 (0)
professional working proficiency	26 (51.0)	20 (52.6)	13 (46.4)
limited working proficiency	13 (25.5)	11 (28.9)	9 (32.1)
elementary proficiency	7 (13.7)	6 (15.8)	6 (21.4)
no proficiency	0 (0)	0 (0)	0 (0)
no response	2 (3.9)	0 (0)	0 (0)
Listening			
native or bilingual proficiency	4 (7.8)	1 (2.6)	1 (3.6)
professional working proficiency	30 (58.8)	24 (63.2)	14 (50.0)
limited working proficiency	10 (19.6)	8 (21.1)	9 (32.1)
elementary proficiency	3 (5.9)	5 (13.2)	4 (14.3)
no proficiency	1 (2.0)	0 (0)	0 (0)
no response	3 (5.9)	0 (0)	0 (0)
Total	51 (100)	38 (100)	28 (100)

Results are shown for each of the three surveys.

For each skill (reading, writing, speaking and listening), the post-course distribution was evaluated using a Shapiro–Wilks test, which showed no violation of normality: reading F(24)* *=* *0.93, *P *=* *0.08; writing F(18) =* *0.90, *P *=* *0.06; speaking F(21) =* *0.92, *P *=* *0.10 and listening F(25) =* *0.93, *P *=* *0.09.

**Figure 2.**
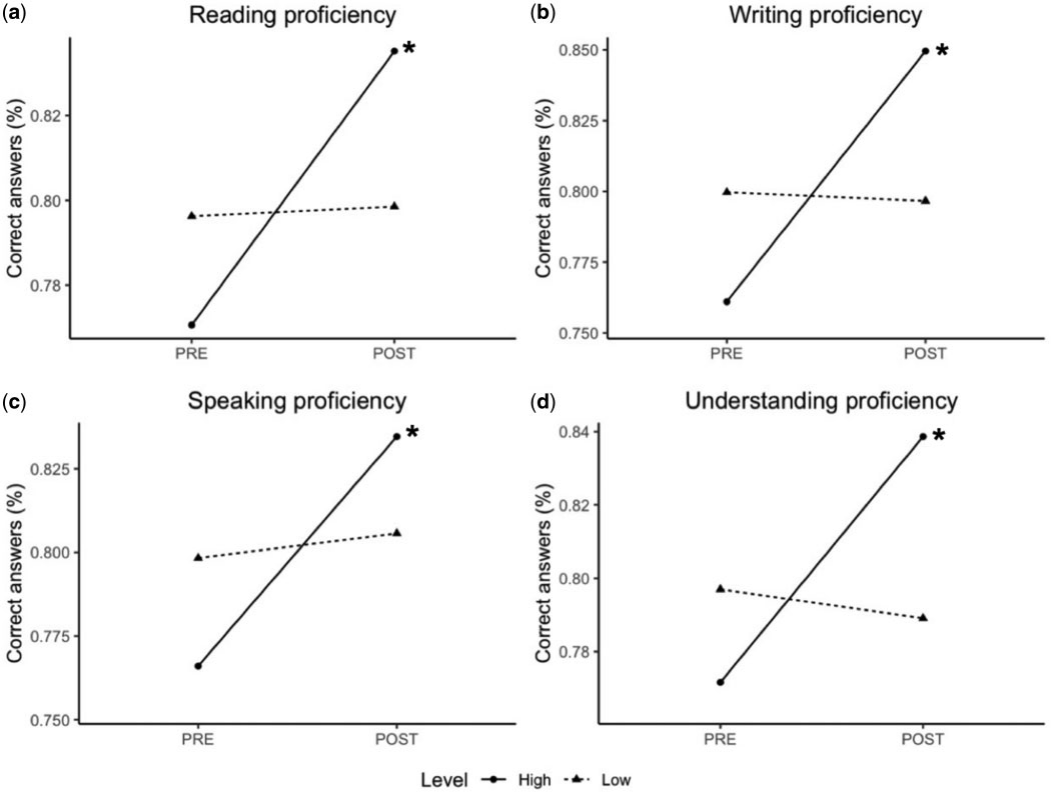
Effect of English proficiency on knowledge improvement. Participants with high English proficiency (solid line) improved significantly during the course, while those with low English level did not (dotted line). Asterisks (*) mark a statistically significant change (*P *<* *0.05) using a one-sample *t*-test.

Participants with a high English level improved significantly during the MOOC (Figure 2). This held true for all four language skills; reading, from 79.7% on the pre-course test to 87.3% on the post-course test (7.6 pp), t(23) =* *3.5, *P *=* *0.002, CI = 0.80–0.87; writing, from 76% to 85% (9 pp), t(17) * *=* *4.6, *P *<* *0.001, CI = 0.81–0.89; speaking, from 77% to 83% (6 pp), t(20) =* *3.5, *P *=* *0.002, CI = 0.79–0.88; and listening, from 77% to 84% (7 pp), t(24) =* *4.0, *P *<* *0.001, CI = 0.80–0.87. As shown by all four dotted lines in Figure 2, no significant knowledge improvement was seen in the groups reporting a low English proficiency.

In the satisfaction survey, two questions considered participants’ own experience of a potential language barrier. Of 28 participants, half disagreed with the statement that ‘taking the course in English created a barrier to understanding the course content’, while over one-third agreed it was a barrier. Furthermore, 53% of respondents agreed they ‘would have benefitted more from the course had it been delivered in their native language’ (29% disagreed).

### Course evaluation

**Figure 3.**
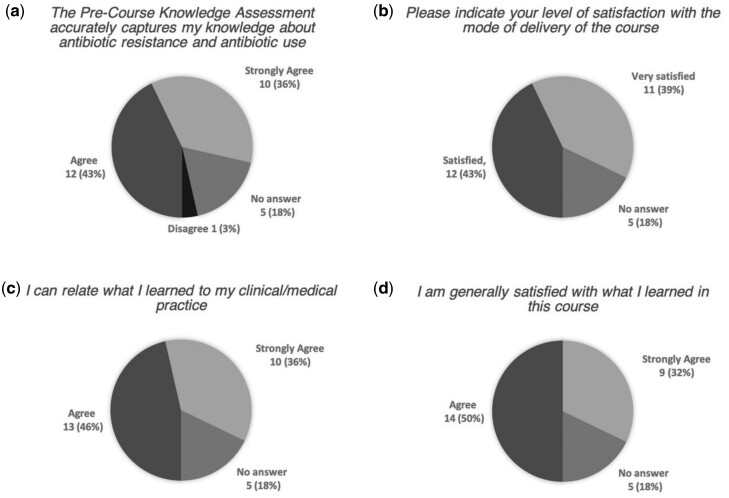
Course satisfaction. For (a), (c) and (d) alternatives were ‘strongly agree’, ‘agree’, ‘disagree’ and ‘strongly disagree’. For (b) alternatives were ‘very satisfied’, ‘satisfied’, ‘dissatisfied’, ‘very dissatisfied’ and ‘not applicable’*.* Five participants (18%) who did not complete the survey are shown as ‘no answer’.

Twenty-eight people filled out the satisfaction survey. Five people (18%) dropped out after initiating the survey and did not provide answers to the succeeding questions—they are shown as ‘no answer’ in Figures 2 and 3. Respondents were generally satisfied with the course and the knowledge assessments (Figure 3).

### Intention to change behaviour

**Figure 4.**
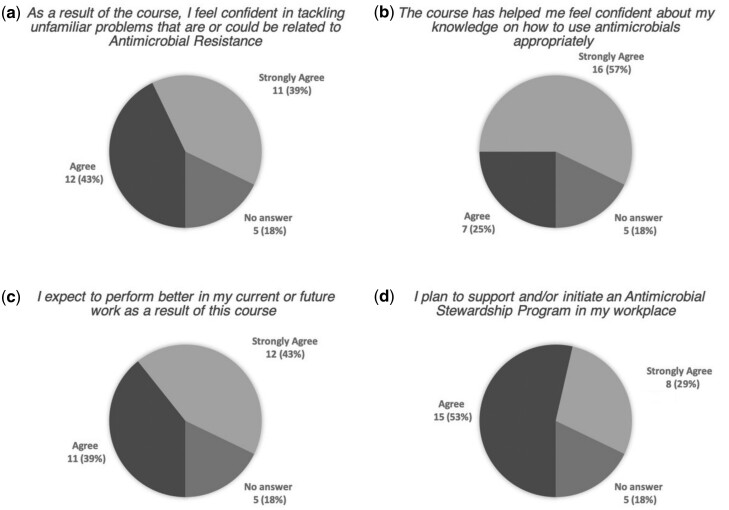
Participants’ evaluation of study impact. Alternatives were ‘strongly agree’, ‘agree’, ‘disagree’ and ‘strongly disagree’. Five participants (18%) who did not complete the survey are shown as ‘no answer’.

Participants estimated the likelihood of changing prescribing behaviour in the question: ‘How likely are you to change your daily clinical practice after taking this course?’ Seventy-five percent of respondents deemed themselves likely or very likely to change their behaviour. Seven percent did not expect to modify their clinical practice. No participant responded ‘not at all’. Further, responses were positive or very positive regarding the study’s impact on confidence in future work capacity, as well as intention to initiate or support AMS programmes (Figure 4).

## Discussion

This study explores the utility of a MOOC to educate in AMS and to facilitate behaviour change among health professionals. To our knowledge, this study was the first to include health professionals from a middle-income country, Macedonia, thereby broadening the geographical context of the research in AMS training. Participants significantly improved their knowledge, but the increase was small. Those who had a high level of self-reported English proficiency made larger knowledge improvements, while those with a lower English level showed no significant improvement—indicating a language barrier. When evaluating the course, participants were satisfied and reported plans to change their behaviour in clinical practice. As this was independent of the measured knowledge improvement, the intention to change behaviour seems to be influenced by other factors in the intervention.

### Knowledge improvement

As the study sample was non-random, the measured knowledge level should not be generalized. It is possible that people who are inclined to participate in an online course on AMS may have a higher pre-understanding of the topic than those who are less willing to partake.

During the course, knowledge significantly improved. The improvement was positively related to prior knowledge in a linear relationship. Scores improved most in the test section with the lowest pre-understanding (67% correct answers), while the section with the highest pre-understanding (99.3% correct answers) showed no improvement. Taking this into account, it may be wise to consider measuring knowledge before conducting education interventions, as it unravels the potential benefits to be made. Thus, modules that participants already master may be excluded and the course may be individualized to only include topics where knowledge is lacking.

### Language barrier

As hypothesized in earlier studies,^18^ a language barrier was found to hinder learning among those with a lower English level. This held true for all four skills measured: reading, writing, speaking and listening. These effects should be seen as four perspectives of the same phenomenon, as English levels over these domains were highly correlated. Hence, it may be sufficient for future studies to simply measure one of the four skills, as they all independently predict knowledge improvement.

### Behaviour change

Previous research has established that a person’s intention to change behaviour is a good predictor of subsequent behaviour change.^28^^,^^29^ To add validity, a measurement of the participants’ estimated behaviour was included. Both intention and estimate of future change were high. This indicates that participants both intended to change their clinical practice and estimated a high likeliness that they would accomplish this change. In the following, ‘intention’ is therefore used to denote both the intentions to change behaviour and the estimation of future behaviour.

Interestingly, participants’ knowledge improvement did not seem to reflect intention to change behaviour. As the surveys were anonymized, English level, which was measured in all tests, was used as a proxy to explore this relationship. When asked about intention to change behaviour, almost all participants were positive or very positive. There was no difference between the high or low English level groups. That is to say, we found no support for the idea that knowledge improvement predicts intention to change behaviour. Instead, other factors related to the intervention may be at play in shaping participants’ intentions.

Applying the stages of change theory, a possible explanation for these findings is that participants were at a higher stage of change than presumed. In the early stages—when not being aware of a problem—knowledge is fundamental to raise awareness. However, as participants already had a high knowledge of AMS, it is possible that simply bringing the topic to attention may in itself have had an impact on their intention to change behaviour—a so-called nudge.^34^^,^^35^

Moreover, the concept of self-efficacy may be applied, which has been explained by Bandura.^34^^,^^36^^,^^37^ Participants reported to have increased confidence in their knowledge and in tackling unfamiliar problems related to AMR, which indicates an increase in self-efficacy. As the increased confidence does not seem to be explained by knowledge improvement, it is more likely that it resulted from some other aspect of the study, such as: taking tests, reflecting on one’s own knowledge in AMS or merely signing up to participate in a study on the subject. An increase in self-efficacy is normally reflected by a move towards the next stage of change.^38^ It is feasible that increased self-efficacy influenced participants’ intention to change their behaviour.

Many participants reported limited time as a major explanation for not partaking in the whole study. For participants with some prior knowledge in AMS, our findings suggest that the behavioural outcome may be reached with a less time-consuming design. More tentative, the findings present the possibility that simply signing up for the course and taking the test may in itself impact self-efficacy and intention to change behaviour. These hypotheses should be tested by future research.

### Dropout

High dropout rates are a core problem for online courses in general.^39^ Compared with similar studies, the dropout rates in the current study were surprisingly low.^15^ A distinctive feature was that participants were recruited through their institutions. This may have raised the threshold for entering the study, indirectly lowering dropout rates. Further, course participants were offered a CME accreditation free of charge if they finished the course, which has been hypothesized to lower dropout.^17^

Anonymization prevented a detailed dropout analysis. As a proxy, participant characteristics in the three surveys were compared, showing no obvious difference regarding gender, workplace, age, medical specialty or English level. That is to say, dropout does not appear to have systematically skewed results. However, no certain conclusion can be made, as non-measured characteristics could have influenced dropouts.

### Limitations and methodological considerations

The non-experimental design without a control group is vulnerable to several threats to internal validity, such as a history threat, a Hawthorne effect, instrumentation or testing threats, or the act of providing the same test twice. These threats are somewhat controlled by the fact that the low English-level group showed no improvement at the second test. It is unlikely that the aforementioned threats systematically handicapped participants from this group. However, the design does not allow us to rule out this possibility.

A second limitation was anonymization and dropouts. Without these, the effect of individual differences could more readily have been identified and statistical power would have been higher.

### Recommendations

The study results indicate two parallel paths for future research and AMS programme design. Firstly, education may be seen as a means to reach the primary outcome: facilitating behaviour change. From this perspective, it is possible that minimizing workload for participants may increase completion rates without reducing effects on behaviour change. To optimize efficacy of AMS programmes, future education interventions should consider measuring pre-course knowledge and, thereafter, design the course to only include parts that fill knowledge gaps. This would allow time-limited health professionals to fully participate. Future AMS research should also investigate the effect of condensed interventions, such as merely inviting participants to take a knowledge test. Building on concepts such as self-efficacy, those interventions may result in effects on behavioural change intention as large as were found in the current study.

Second, when education was seen as the main outcome, our study found support for a language barrier hindering knowledge improvement. Physicians with a self-reported high English level did benefit from the course, while those with a lower level did not. In order to permit a global spread of AMS online education, the material needs to be provided in the native language of health professionals.

## Supplementary Material

dlaa045_Supplementary_Data
