# Supplementary material for: Building antimicrobial stewardship through massive open online courses: a pilot study in Macedonia
Source: JAC Antimicrob Resist. 2020 Jul 18;2(3):dlaa045. doi: 10.1093/jacamr/dlaa045 (PMC8210003; doi:10.1093/jacamr/dlaa045)
Supplement: dlaa045_Supplementary_Data [file dlaa045_supplementary_data.zip › Supplementary_data.docx]

Supplementary data

Appendix 1.

PRE-COURSE AND POST-COURSE KNOWLEDGE ASSESSMENT

**Survey and answer sheet**

## Main category I: Awareness and Knowledge

### This main category on *Awareness and Knowledge* includes questions aiming at assessing your prior knowledge about bacteria, antibiotics, bacterial resistance, and infection prevention and control.

Section 1.1 Knowledge about Bacteria *(5 questions)*

#### For each of these questions, please tick (✓) the box next to the answer of your choice.

1. Which of the following bacteria is most prevalent in cat and dog bite infections? (Select one)
   - Group A streptococcus
   - *Pasteurella multocida*
   - Bacteria of the Enterobacteriaceae family
   - Clostridium dificile
2. Which of the following bacteria can form spores? (Select one)
   - Staphylococci
   - *Clostridia*
   - Escherichia coli (E. coli)
   - Listeria monocytogenes
3. Which of the following bacteria occur in unpasteurized products? (Select one)
   - Staphylococcus saprophyticus
   - Pasteurella multocida
   - *Listeria monocytogenes*
   - Bordatella pertussis
4. Which of the following bacteria have no cell wall? (Select one)
   - Streptococcus pneumonia
   - *Mycoplasma pneumonia*
   - Staphylococcus aureus
   - Mycobacterium tuberculosis
5. Which of the following bacteria is normally not part of the intestinal flora? (Select one)
   - *Staphylococcus aureus*
   - Enterococci
   - Escherichia coli (E. coli)
   - Shigella flexneri

### Section 1.2 Knowledge about Antibiotics

#### For each of these questions, please tick (✓) the box next to the answer(s) of your choice.

1. Alexander Fleming discovered the first antibiotic in 1928. What was the antibiotic named? (Select one)
   - Mold
   - *Penicillin*
   - Vancomycin
   - Doxycycline
2. Which of these illnesses should be treated with antibiotics? (Select one)
   - Rhinovirus
   - Influenza
   - *Streptococcal Throat infection*
   - Mononucleosis
3. Antibiotics are effective against colds and flu (Select one)
   - *False: colds and flu are mostly caused by viruses, which antibiotics do not work against*
   - Partly correct: antibiotics sometimes work against viruses
   - True: antibiotics work against everything
   - Partly correct: antibiotics are effective against flu, but not colds
4. Which of the following antibiotics is a beta-lactam antibacterial drug? (Select one)
   - Metronidazole
   - Clindamycin
   - *Cloxacillin*
   - Trimethoprim/Sulfametoxazole
5. Which of the following antibiotics have a bactericidal effect? (Select one)
   - *Amoxicillin/Clavulanic*
   - Doxycycline
   - Clindamycin
   - Trimethoprim/Sulfametoxazole
6. Which of the following antibiotics is **not** used to treat pneumonia caused by atypical pathogens? (Select one)
   - Tetracyclines
   - Erythromycin
   - *Amoxicillin/Clavulanic*
   - Clarithromycin
7. Beta-lactam antibiotics display which of the following mechanisms of action? (Select one)
   - Inhibits the protein synthesis of the bacterium
   - *Inhibits cell wall synthesis*
   - Inhibits the bacterial folic acid synthesis
   - Inhibits the bacterial DNA synthesis
8. Why is phenoxymethylpenicillin (also known as Penicillin V) ineffective in the treatment of mycoplasma pneumonia? (Select one)
   - *Mycoplasma has no cell wall*
   - Mycoplasma is a virus
   - Mycoplasma form beta-lactamases that degrades penicillin
   - Mycoplasma is a so-called *superbug*
9. Which of the following conditions should generally not be treated with antibiotic therapy in normal hosts? (Select one)
   - Streptococcal pharyngitis
   - *Asymptomatic bacteriuria*
   - Sepsis
   - Pyonephritis
10. Broad spectrum antibiotic use contributes to the development of Clostridium difficile infection:
    - *True*
    - False
11. Which of these would be suitable to treat bacteremia due to gram-positive cocci (Select all that apply)?
    - *Cloxacillin*
    - *Vancomycin*
    - Ciprofloxacin
    - Trimethoprim
    - Ganciclovir
12. Gentamicin dosing is based on actual body weight and therefore obese patients will need a significantly higher dose than lean patients:
    - True
    - *False*
13. Outpatient parenteral antibiotic therapy (OPAT*) using intravenous antibiotics therapy could be useful for the treatment of some cases of osteomyelitis:
    - *True*
    - False
14. All of the following conditions could be referred to an outpatient parenteral antibiotic therapy (OPAT*)-team except? *(Select one)*
    - Resolving cellulitis needing a further 7 days therapy
    - An extended-spectrum beta-lactamase positive urinary tract infection
    - *Meningitis – from day 2 of therapy*
    - Osteomyelitis needing a further 6 weeks of treatment

Section 1.3 Understanding of Bacterial Resistance *(15 questions)*

#### For each of these questions, please tick (✓) the box next to the answer(s) of your choice.

1. Which of the following statements explains the spread of antibiotic-resistant strains of bacteria: (Select one)
   - *antibiotic-resistant strains have a selective advantage in the presence of the antibiotic(s) to which they are resistant*
   - antibiotic-resistant strains have better genes
   - antibiotic-resistant strains do not grow in the presence of the antibiotic
   - antibiotic-resistant strains are toxic
2. In the classification of antimicrobial resistance, what does MRSA stand for? (Select one)
   - Medically resistant Staphylococcus aureus
   - Methicillin-resistant Streptococcus antibodies
   - Multiple resistant Staphylococcus aureus
   - *Methicillin-resistant Staphylococcus aureas*
3. Which of the following can be used for empirical treatment in suspected cases of MRSA? (Select one)
   - Isoxazolyl penicillin
   - *Vancomycin*
   - Cephalosporins
   - Ampicillin
4. In the classification of antimicrobial susceptibility, what does the acronym SIR stands for? (Select one)
   - *S = susceptible; I = intermediate; R = resistant*
   - S = susceptible; I = indeterminable; R = resistant
   - S = sensorineural; I = intermediate; R = resistant
   - S = susceptible; I = intermediate; R = refractory
5. Which is the most common notifiable resistance? (Select one)
   - *Extended-spectrum beta-lactamases (ESBLs)*
   - Multi-drug resistant Tuberculosis (MDR-TB)
   - Vancomycin-resistant enterococci (VRE)
   - Carbapenem-resistant Enterobacteriaceae infection (CRE)
6. Which of the following drug classes are usually successful in the treatment of ESBL- producing bacteria? (Select one)
   - Cephalosporins
   - Clindamycin
   - *Carbapenems*
   - Amino-Penicillins
7. Which of the following is not an effective intervention in preventing the emergence or spread of antibiotic resistant pathogens? (Select one)
   - Adherence to hand hygiene
   - Contact isolation during hospitalization for patients colonized with Methicillin- resistant Staphylococcus aureus
   - Avoiding the use of antibiotics for viral infections
   - *Treating infections for a longer duration*
8. Which of the following is not a current example of clinically important antibiotic resistance? (Select one)
   - Meticillin-resistant Staphylococcus aureus
   - *Penicillin-resistant Streptococcus pyogenes (Group A Strep)*
   - Fluoroquinolone-resistant Pseudomonas aeruginosa
   - Vancomycin-resistant Enterococci
9. Which of the following is ***not*** a way that a bacterium can acquire antibiotic resistance?

*(Select one)*

- - *Acquiring resistance genes from host cells*
  - Through spontaneous mutation
  - From its parent cell
  - Scavenging resistance genes from the environment
  - Exchanging DNA with another bacterium

1. If an infection is resistant to ciprofloxacin, parenteral treatment with an alternative drug is the only option:
   - *True*
   - False
2. Extended-spectrum beta-lactamase-producing organisms may be resistant to common antibiotics including those without a beta-lactam ringed structure:
   - *True*
   - False
3. What can happen if a patient gets an antibiotic-resistant infection? (Select all that apply)
   - *He/she may have a longer-lasting illness*
   - *He/she may require hospitalization*
   - *He/she may need more costly medicine to treat the infection*
   - *He/she may experience more side effects*
4. Antibiotic resistance has been called one of the world's most pressing public health problems
   - *True*
   - False
5. Rising rates of antibiotic resistance cannot be reversed by changes in antibiotic prescribing practices
   - True
   - *False*
6. New antibiotics are being developed at an increasing rate in order to combat antibiotic resistant bacteria
   - True
   - *False*

### Section 1.4 Understanding of Infection Prevention and Control *(5 questions)*

#### For each of these questions, please tick (✓) the box next to the answer of your choice.

1. Which are the fundamental elements needed to prevent transmission of infectious agents in healthcare settings (Select all that apply)?
   - *Hand hygiene*
   - *Surveillance for healthcare-associated infections*
   - *Clinical microbiology laboratory support*
   - *Adherence of healthcare personnel to recommended infection prevention and control guidelines*
   - *Education on the principles and practices for preventing transmission of infectious agents*


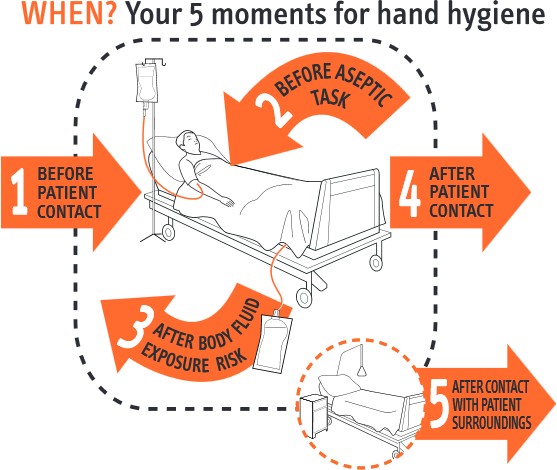
Hand hygiene:

1. Hand hygiene must be performed in all indications described (in the image above) regardless of whether gloves are used or not.
   - *True*
   - False
2. So called “Standard Precautions” are meant to reduce the risk of transmission of bloodborne and other pathogens from both recognized and unrecognized sources
   - *True*
   - False
3. Key elements of “Standard Precautions” are (Select all that apply)
   - Hand hygiene
   - Use of personal protective equipment guided by risk assessment
   - Respiratory hygiene
   - Environmental control (cleaning and disinfection) and safe waste management
   - Cleaning and disinfection of medical equipment
   - Prevention of needle stick and injuries
4. Early recognition of infection, prompt implementation of Infection Prevention and Control (IPC) precautions, reporting and surveillance, as well as treatment of the infection, are all important methods to reduce and prevent dissemination of infectious agents in the healthcare setting:
   - *True*
   - False
5. Healthcare-associated infections (HAIs) can result in serious illness, prolonged hospital stay, excessive use of health-care resources, and may result in loss of life.
   - *True*
   - False

## Main category II: Prescribing competency

### The second main category (II) includes two sections with questions aiming at assessing your knowledge about best practice in patient safety and diagnostics.

Section 2.1 Patient Safety

#### For each of these questions, please tick (✓) the box next to the answer of your choice.

1. Which of the following antibacterial drugs can be given to pregnant women? (Select all that apply)
   - Ciprofloxacin
   - Doxycycline
   - *Penicillin*
   - Metronidazole
2. Which of the following antibacterial drugs can preferably be given to children? (Select one)
   - Ciprofloxacin
   - *Isoxazolyl penicillin*
   - Tetracyclines
   - Azithromycin
3. Which of the following antibacterial drugs is the most nephrotoxic? (Select one)
   - *Gentamicin*
   - Cephalosporin
   - Clindamycin
   - Metronidazole
4. What is the nearest approximate percentage figure for the proportion of patients with penicillin allergy who may also be allergic to cephalosporin? (Select one)
   - *0.5-6.5%*
   - 5-10.5%
   - 30%
   - 50%
5. Which antibiotic drug(s) can be administered to a patient who had an anaphylactic reaction to penicillin without a concern for cross-reactivity? (Select all that apply)
   - Cefuroxime
   - *Gentamicin*
   - *Ciprofloxacin*
   - Meropenem
   - *Clarithromycin*
6. Trimethoprim is associated with a drug interaction that could be life threatening
   - *True*
   - False
7. How often do you check prescribing guidelines, when you prescribe antibiotics? (Select one)
   - Always
   - Regularly
   - Occasionally
   - Never
8. How confident are you that your own prescribing of antibiotics follows the best available evidence?
   - Very confident
   - Somewhat confident
   - Somewhat unsure
   - Unsure

*For each of the following statements, please indicate your level of agreement.*

| 49. | In my place of work, antibiotics are overused | Strongly Agree | Agree | Disagree | Strongly Disagree |
| --- | --- | --- | --- | --- | --- |
| 50. | In my place of work, antibiotic resistance is no problem | Strongly Agree | Agree | Disagree | Strongly Disagree |
| 51. | Prescribing antibiotics does not cause harm, even if the patient does not really need them | Strongly Agree | Agree | Disagree | Strongly Disagree |

| What proportion of antibiotic prescribing do you consider to be unnecessary when it comes to: | | | | | |
| --- | --- | --- | --- | --- | --- |
| 52. | Your own prescribing | <10% | 11-20% | 21-50% | >50% |
| 53. | Your division’s prescribing (e.g., at the hospital ward) | <10% | 11-20% | 21-50% | >50% |
| 54. | The overall level of prescribing (e.g. at the hospital) | <10% | 11-20% | 21-50% | >50% |

### Section 2.2 Diagnostics and Indication

#### For each of these questions, please tick (✓) the box next to the answer of your choice.

1. In the classification of antimicrobial susceptibility, what does the acronym MIC stands for? (Select one)
   - Minimum inflammatory CRP
   - Maximum inhibitory concentration
   - *Minimum inhibitory concentration*
   - Maximum inhibitory CRP
2. Which of the following urinary tract pathogens do not form nitrite detectable on a urine dipstick? (Select one)
   - Klebsiella pneumonia
   - *Staphylococcus saprophyticus*
   - Escherichia coli (E. coli)
   - Proteus mirabilis
3. How do you interpret a disk diffusion test? (Select one)
   - A small zone of inhibition means that the bacterium is more sensitive to this antibiotic
   - *A big zone of inhibition means that the bacterium is more sensitive to this antibiotic*
   - No zone of inhibition means that the bacterium is more sensitive to this antibiotic
   - A big zone of inhibition means that the bacterium is less sensitive to this antibiotic
4. How do you interpret a minimum inhibitory concentration (MIC)-value? (Select one)
   - The higher the value the more sensitive is the bacterium to this antibiotic
   - *The lower the value the more sensitive is the bacterium to this antibiotic*
   - The higher the value the narrower the spectrum of the antibiotic
   - The lower the value the broader the spectrum of the antibiotic
5. For which of the following antibacterial drugs is the time above the minimum inhibitory concentration (MIC) decisive for the treatment effectiveness? (Select one)
   - Clindamycin
   - Nitrofurantoin
   - *Cloxacillin*
   - Vancomycin
6. Which of the following blood culture results most likely represents a contamination and should not automatically be treated with antibiotics? (Select one)
   - One of two blood culture bottles positive with Group A Streptococci
   - One of two blood culture bottles positive with a gram-negative bacterium
   - *One of two blood culture bottles positive with coagulase-negative Staphylococci*
   - One of two blood culture bottles positive with Klebsiella species
7. Absorption of rectal vancomycin is insignificant and monitoring blood levels is unnecessary
   - True
   - *False*
8. Which of these conditions is an indication for therapy? (Select one)
   - A catheter specimen urine of a stroke patient positive with > 1 X 10E5 Colony- forming unit (CFU) /ml Candida species
   - A catheter specimen urine of a patient with heart failure, positive with > 1 X 10E5 Colony-forming unit ^(^CFU)/ml Coliforms
   - *Repeat isolation in a catheter specimen of urine of >* 1 X 10E5 *Candida species in an immunosuppressed patient*
   - A catheter specimen urine of a patient after hip replacement surgery positive with > 1 X 10E5 Colony-forming unit (CFU) /ml Coliforms

***Main category III: Managing Infections (5 questions)***

### This is the last main category (III) and includes questions aiming at assessing your prior knowledge about managing infections.

#### For each of these questions, please tick (✓) the box next to the answer(s) of your choice.

1. If appropriate and indicated by clinical evidence the following strategies can be applied to improve antibiotic use (Select all that apply)
   - *Stop treatment*
   - *Switch from intravenous (IV) to oral antibiotics*
   - *Change spectrum to narrower spectrum if possible*
   - *Review treatment again after 72 hours after treatment initiation*
   - *Switch to outpatient parenteral antibiotic therapy (OPAT)*
2. At what point should an “antibiotic timeout*” be taken in a patient receiving empiric antibiotics (according to the CDC)? (Select one)
   - 5-7 days after empiric therapy is started
   - 24 hours after empiric therapy is started if supporting clinical and microbiologic data is available
   - 48-72 hours after empiric therapy is started
   - *24 hours after empiric therapy is started if supporting clinical and microbiologic data is available + 48-72 hours after empiric therapy is started*
   - Never
3. Which of the following could be a result of an “antibiotic timeout*”? (Select one)
   - *De-escalation of antibiotics*
   - *Cessation of antibiotics*
   - *Escalation of antibiotics*
   - *No change*
4. Which of the following are relevant factors in considering de-escalation or adjusting antibiotic therapy during an “antibiotic timeout*”? (Select all that apply)
   - *Antibiotic susceptibility of bacteria from culture*
   - Results from urine dip-stick test
   - *Organ site of identified infection*
   - *Changes in biomarkers of infection (such as procalcitonin)*
   - *Clinical status of the patient*
5. A 64-year old woman was admitted to hospital because of syncope and a cardiac arrhythmia. A subclavian central venous catheter is placed and she is continuously monitored. On day 3 she underwent cardiac electrophysiology studies. On day 4 of hospitalization, she developed fever and leukocytosis. Chest Xray was clear and urinalysis was normal. Blood cultures were obtained, the central venous access catheter was removed, and empiric antibiotic therapy (vancomycin and levofloxacin) was started. On day 5, the laboratory reports that the blood cultures are all growing a staphylococcus and on the following day, they report that it is susceptible to cloxacillin and to cephalosporins. Which among the following is the optimal choice regarding this patient's antibiotic therapy? (Select one)
   - Continue vancomycin and levofloxacin
   - Continue vancomycin, discontinue levofloxacin
   - *Discontinue vancomycin and levofloxacin, start cloxacilin*
   - Continue vancomycin, discontinue levofloxacin, add cloxacilin

# **Thank you for your participation in this survey!**

**Appendix 2.**

**PARTICIPANT SATISFACTION SURVEY**

**Personal background information**

### 1. What is your gender?

- Female
- Male

### 2. What is your age?

- 18 to 24
- 25 to 34
- 35 to 44
- 45 to 54
- 55 to 64
- 65 to 74
- 75 or older

### 3. What is your nationality?

### ___________________________________

### 4. What is your first language?

### ___________________________________

### 5. What is your medical specialty?

### ___________________________________

### 6. Please indicate your level of English language proficiency

|  | **No proficiency** | **Elementary proficiency** | **Limited working proficiency** | **Professional working proficiency** | **Native or bilingual proficiency** |
| --- | --- | --- | --- | --- | --- |
| Read |  |  |  |  |  |
| Write |  |  |  |  |  |
| Speak |  |  |  |  |  |
| Understand |  |  |  |  |  |

### 7. To what extent have your expectations for this course been met? (Select one)

- Fully
- Partially
- Barely
- Not at all

### 8. Have you attempted to take the Post-Course Knowledge Assessment?

- Yes
- No

### 9. Have you also taken the exam for CME credit?

- Yes
- No

**General questions**

**Section 1: Expectations, Completion, and Non-Completion**

### 10. If you did not attempt to take the exam, what is (are) the reason(s)?(Select all that apply)

I was busy with my work

- I lost interest in the course
- The study demands were too high
- I felt I was not properly prepared for the exam
- Other (please specify) _____________________________

### 11. If you attempted to take the exam for CME credit, how many attempts were used (out of 3 total)?

- 1
- 2
- 3 (maximum number of attempts)
- Not applicable, I did not attempt to take the exam

### 12. Did you successfully pass the exam for CME credit?

- Yes
- No
- Don't know
- Not applicable, I answered "No" to the previous question

### 13. What proportion of the video lectures did you watch?(Select one)

- All of them
- More than half
- About half
- Less than half
- None

**Questions for participants who did not fully complete the Course (Q14-15)**

**Non-Completion**

### 14. If you watched about half of the lectures or less, what is (are) the reason(s)?(select all that apply)

- I was busy with my work
- I lost interest in the course
- The study demands were too high
- The course did not meet my expectations
- Other (please specify) _____________________________

### 15. “I would have been more likely to complete the course, if..."

|  | Strongly agree | Agree | Disagree | Strongly disagree | Not applicable |
| --- | --- | --- | --- | --- | --- |
| I had more than 4 weeks’ time to complete it |  |  |  |  |  |
| The pacing of the lecture presentations was slower |  |  |  |  |  |
| The pacing of the lecture presentations was faster |  |  |  |  |  |
| The course were structured in a different way |  |  |  |  |  |
| The course content were different |  |  |  |  |  |
| The course were delivered using different technology |  |  |  |  |  |
| I could download the lecture slides (e.g. as powerpoint or pdf) |  |  |  |  |  |
| I were able to interact with other course participants |  |  |  |  |  |
| I were able to interact with the lecturers |  |  |  |  |  |
| it was delivered onsite/face-to-face, rather than online |  |  |  |  |  |

**Section 2: Satisfaction**

### * 16. Which delivery mode do you prefer for a course? (Select one)

- All online (Video lectures, webinars, teleconference, discussion forums, etc.)
- Majority online
- 50/50 (online/face-to-face) Majority face-to-face
- All face-to-face

### 17. How much time did you spend on the course in total (including video lectures, reading the literature, exam, and surveys)? (Select one)

- More than 20 hours
- 10-15 hours
- 5-10 hours
- Less than 5 hours

### 18. How would you rate the course length/total time? (Select one)

- Much too long
- Slightly too long
- Just right
- Slightly too short
- Much too short

### 19. How would you rate the pacing of this course? (Select one)

- Much too slow
- Slightly too slow
- Just right
- Slightly to fast
- Much too fast

### 20. Please rate the overall teaching abilities of the speakers in this course (Select one)

- Excellent
- Very good
- Moderate
- Poor

### * 21. How would you rate the difficulty of this course? (Select one)

- Much too difficult
- Slightly too difficult
- Just right
- Slightly too easy
- Much too easy

### 22. Please indicate your level of satisfaction with the following elements of the course

|  | **Very satisfied** | **Satisfied** | **Dissatisfied** | **Very dissatisfied** | **Not applicable** |
| --- | --- | --- | --- | --- | --- |
| The instructions you received in advance of taking the course |  |  |  |  |  |
| The supportive resources available, like course bibliography, references etc. |  |  |  |  |  |
| Selection of lecture topics |  |  |  |  |  |
| Sequence of the topics (order of the lectures, etc.) |  |  |  |  |  |
| Overall course content |  |  |  |  |  |
| Overall course structure |  |  |  |  |  |
| Mode of delivery of the course |  |  |  |  |  |
| Consistency of the course content with the course objectives as stated in the Course overview |  |  |  |  |  |
| The Guidelines the course is based on (Infectious Disease Society of America (IDSA) Guidelines) |  |  |  |  |  |
| Digital pad lecturing  *(the writing appears while the lecturer is speaking)* |  |  |  |  |  |
| Power Point lecturing  *(slides are shown while the lecturer is speaking)* |  |  |  |  |  |
| On-demand availability of the lecture videos |  |  |  |  |  |
| Opportunity to stop, pause and/or rewind lectures |  |  |  |  |  |
| The pre-course knowledge assessment |  |  |  |  |  |
| The post-course knowledge assessment |  |  |  |  |  |
| The exam for CME credit |  |  |  |  |  |

### * 23. Please indicate your level of agreement with the following statements

|  | **Strongly agree** | **Agree** | **Disagree** | **Strongly disagree** | **Not applicable** |
| --- | --- | --- | --- | --- | --- |
| I was able to manage features of the learning platform (i.e. video files, online surveys, course website, etc.) |  |  |  |  |  |
| I am generally satisfied with what I learned in this course |  |  |  |  |  |
| My learning experience was enhanced by the on-demand availability of the lectures |  |  |  |  |  |
| The lectures were clear and easy to understand |  |  |  |  |  |
| I had difficulty in understanding what the speakers said because of technical reasons (sound quality, streaming speed, etc.) |  |  |  |  |  |
| Taking this course in English created a barrier to understanding the course content |  |  |  |  |  |
| I would have benefitted more from the course had it been delivered in my native language |  |  |  |  |  |
| I would have benefitted more from the course had this course been based on my national treatment guidelines |  |  |  |  |  |

### * 24. Please indicate your level of agreement with the following statements

|  | **Strongly agree** | **Agree** | **Disagree** | **Strongly disagree** | **Not applicable** |
| --- | --- | --- | --- | --- | --- |
| This course improved my understanding of Antibiotic Stewardship |  |  |  |  |  |
| The course content was appropriate for the target audience (physicians) |  |  |  |  |  |
| The course content was relevant to the Antimicrobial resistance (AMR) situation in my country |  |  |  |  |  |
| I can relate what I learned to my clinical/medical practice |  |  |  |  |  |
| During the course I was able to apply my previous knowledge and experiences |  |  |  |  |  |
| I would like to take more advance courses on this topic |  |  |  |  |  |
| I would have done better in the course if there were opportunities to interact with the faculty |  |  |  |  |  |

**Knowledge Improvement**

### 25. How would you rate your knowledge improvements in the following areas as a result of participating in the course

|  | **Excellent** | **Very good** | **Average** | **Poor** |
| --- | --- | --- | --- | --- |
| Burden of antimicrobial resistance in my country |  |  |  |  |
| What I can do as a prescriber to influence antimicrobial resistance |  |  |  |  |
| Main drivers of antimicrobial resistance in hospitals |  |  |  |  |
| Types of Antimicrobial Stewardship Interventions |  |  |  |  |
| Measures for infection prevention and control (IPC) |  |  |  |  |
| Proper use of antibiotics |  |  |  |  |
| Role of laboratory diagnostics in determining an appropriate treatment |  |  |  |  |
| Antimicrobial Stewardship interventions for specific medical disciplines |  |  |  |  |

**Section 3. Personal benefits from taking this course**

### 26. Please indicate your level of agreement with the following statements

|  | **Strongly agree** | **Agree** | **Disagree** | **Strongly disagree** |
| --- | --- | --- | --- | --- |
| The course was intellectually stimulating |  |  |  |  |
| The course has helped me feel confident about my knowledge on how to use antimicrobials appropriately |  |  |  |  |
| As a result of the course, I feel confident in tackling unfamiliar problems that are or could be related to Antimicrobial Resistance |  |  |  |  |
| I have reached my personal goals of this course |  |  |  |  |

**Section 4. Personal future plans**

### 27. How likely are you to change your daily clinical practice after taking this course? (Select one)

- Very likely
- Likely
- Not very likely
- Not at all

### * 28. Please indicate your level of agreement with the following statements

|  | **Strongly agree** | **Agree** | **Disagree** | **Strongly disagree** |
| --- | --- | --- | --- | --- |
| I expect to perform better in my current or future work as a result of this course |  |  |  |  |
| I plan on mentioning my participation in this course to current or future employers |  |  |  |  |
| Earning Continuing Medical education (CME) credit was a significant motivating factor for taking this course |  |  |  |  |
| Earning a certificate was a significant motivating factor for taking this course |  |  |  |  |
| I plan to support and/or initiate a Antimicrobial Stewardship Program in my workplace |  |  |  |  |

### 29. Please share any recommendations for improvement of this course

### __________________________________________________________

### 30. Please indicate your level of agreement with the following statements

|  | **Strongly agree** | **Agree** | **Disagree** | **Strongly disagree** |
| --- | --- | --- | --- | --- |
| Taking the Pre-Course Knowledge Assessment was a good preparation for the course |  |  |  |  |
| The Pre-Course Knowledge Assessment accurately captures my knowledge about antibiotic resistance and antibiotic use |  |  |  |  |
| More quizzes and/or exercises throughout the course would enhance my learning experience |  |  |  |  |
| Feedback from the professor on the final exam would enhance my learning experience |  |  |  |  |
| The possibility to discuss and exchange with fellow course participants would enhance my learning experience |  |  |  |  |

### 31. If you would like information on future opportunities for online education, please leave your e-mail address below

_____________________________________________________________________

# Thank you for your participation in this survey!

Your responses will help us to adapt and improve the online course “Antibiotic Stewardship: Optimization of Antibiotic Practices” and other related online education material.
